# The Morrison Engine Infringement

**Published:** 1874-03

**Authors:** 


### THE MORRISON ENGINE INFRINGEMENT.

The indications are, just now, that there will be "quite a stir in the dental profession ere long in reference to the Morrison Engine." It is claimed that this very popular appliance is an infringement upon a patent issued in 1866 for a machine devised and constructed for running sheep shears.

This machine appears to possess all the principles of the Morrison Engine, except the hand piece, and undoubtedly by the date of its patent has precedence. Why does the patent department issue two patents for the same thing, or for two machines involving the same principle?

A demand is now made upon all who are now using the engine, that a license be obtained from the original patentee for each and every engine in use; the charge for which is $25.

We do not propose to give advice in this matter, but we will not take out a license nor pay the fee for the present at least.

If the patentee or the manufacturers of the Morrison Engine had any knowledge of this former patent, they did great injustice to themselves and to the profession by introducing that which would cause trouble, perhaps litigation, and hard feeling. But, if they had no such knowledge then, they now have, and they should proceed at once to make such arrangements with the owner of the former patent as will protect all those who are using or may use the Morrison Engine. This is justice; this much, and not less, in this matter we ask.

